# Meaningful work experiences of certified primary care physicians in Japan: a qualitative study

**DOI:** 10.1186/s12875-025-03026-2

**Published:** 2025-10-28

**Authors:** Yu Yamamoto, Junji Haruta, Ryohei Goto, Tetsuhiro Maeno

**Affiliations:** 1 General Medicine and Primary Care, Institute of Medicine, University of Tsukuba, 1-1-1 Tennodai, Tsukuba, Ibaraki 305-8575 Japan; 2 Central General Clinic, 4-58-1 Kamikashiwada, Ushiku, Ibaraki 300-123 Japan; 3 Medical Education Center/Center for General Medicine Education, School of Medicine, Keio University, 35 Shinanomachi Shinjuku-ku, Tokyo, 160-8582 Japan; 4 Department of Primary Care and Medical Education, Institute of Medicine, University of Tsukuba, 1-1-1 Tennodai, Tsukuba, Ibaraki 305-8575 Japan

**Keywords:** Primary care physician, Interview, Meaningful work

## Abstract

**Background:**

The aim of this study was to explore what experiences certified primary care physicians (PCPs) in Japan found meaningful in their work.

**Method:**

Between October 2021 and February 2022, semi-structured interviews were conducted with Japan Primary Care Association (JPCA)-certified family physicians or JPCA Diplomates in primary care who were working at clinics or small hospitals in Japan regarding “work experiences that they felt were meaningful as physician’s work”. The interviews were conducted face-to-face or via video call. The data obtained were transcribed verbatim and data analysis was conducted using inductive thematic analysis.

**Results:**

Fourteen physicians participated in the interview survey. Six themes were identified regarding work experiences that PCPs found meaningful: 1) management of diverse health problems, 2) comprehensive approach to patients, their families, and their issues, 3) trust relationships with patients built by continuity, 4) experience supporting patients with complex problems through interprofessional collaboration, 5) contribution to healthcare provider and medical student education, and 6) contribution to the community and society.

**Conclusion:**

PCPs appear to find meaning in their work through two main pathways. First, they improve their own clinical abilities through practice. Second, they experience fulfillment by contributing to patients and the broader community. This clarification of the specific experiences of PCPs that are related to the meaning of work could potentially encourage PCPs working in various settings.

**Supplementary Information:**

The online version contains supplementary material available at 10.1186/s12875-025-03026-2.

## Background

The development of primary care systems among countries has occurred within the cultural and historical context of each country. In Europe and other countries with advanced primary care, an established primary care system began to emerge in the mid-1900s. Among important milestones, the World Organization of National Colleges, Academies and Academic Associations of General Practitioners/Family Physicians (WONCA) was founded in 1972 across 18 countries and has since grown into the world’s largest academic association for family medicine [1]. This led to the establishment of primary care systems in Western countries, each tailored to its specific national circumstances [2, 3]. In Japan, the need to recruit doctors in primary care has been discussed as a response to the greater demand for primary care due to the low birthrate and aging population [4, 5]. The rapid aging has resulted in more patients presenting with complex, chronic, and psychosocial issues that extend beyond specific organ systems [6], which in turn has increased the need for primary care physicians, who are characterized by the “breadth and diversity of issues they handle.” In this paper, we define ‘primary care (PC)’ and ‘primary care physician (PCP)’ as encompassing family medicine, general practice, and general medicine, along with family physicians and general practitioners.

Until 20 years ago, there were no PC specialists in Japan. Instead, organ-specific specialists provided PC in their own clinics or in small to medium-sized hospitals [7]. The Japan Primary Care Association (JPCA), an academic organization in the field of PC in Japan, launched its own postgraduate training program for PC in 2010 to train PC specialists [7, 8]. In 2018, the Japanese Medical Specialty Board established a new PC specialist system, the Board-Certified Doctor of General Medicine, thereby officially establishing the PC specialist field in Japan [7]. In the field of PC in Japan, clinic physicians continue to provide home visit care in addition to outpatient care. With the exception of some advanced medical institutions, patients can visit hospitals without a referral. This means physicians at small and medium-sized hospitals also provide PC by performing outpatient as well as inpatient and emergency care [6]. In addition, many physicians are involved not only in clinical work but also in education, research, and community activities [9]. Given this context, Japan’s PC specialist training curriculum is unique because it requires physicians to gain experience in both clinics and hospitals [10]. 

With the establishment of this unique PC system, however, PCPs have reported career concerns. These include the lack of a quality assurance system, insufficient career information, absence of a supportive network for sharing ideas and gaining empathy, anxiety about an ambiguous identity, and a scarcity of role models [11, 12]. Research surrounding these kinds of career concerns has generally indicated that experiences making work feel more meaningful tend to increase job satisfaction and promote work engagement, and are negatively associated with anxiety, depression, and intentions to leave the profession [13–15]. Experiences that PCPs in other countries are reported to find meaningful include the diagnosis and treatment of common diseases, care of chronic illnesses, and management of disease risks [16, 17]. Specifically in Japan, PCPs who derive greater positive meaning from their work show more enthusiasm for outpatient care and involvement in research [18]. A qualitative study in which Japanese geriatricians were interviewed about job satisfaction revealed that they enjoyed providing comprehensive primary care and engaging in interprofessional collaboration [19]. Further, they perceived their work as a promising career and expressed satisfaction with their patient communication [19]. However, the specific work experiences that PCPs in Japan find meaningful remain unknown. One definition of meaningful work is “the global judgment that one’s work accomplishes significant, valuable, or worthwhile goals that are congruent with one’s existential values [13].” In this paper, we define “meaningful experience” as an experience that leads to professional development and is perceived by physicians as valuable or positive.

In this study, to identify the specific work experiences that certified PC specialist in Japan find meaningful, we conducted semi-structured interviews with Japan Primary Care Association (JPCA)-certified family physicians or JPCA Diplomates in primary care who were working at clinics or small hospitals in Japan. Given the diverse tasks and settings that Japanese PCPs navigate, identifying and sharing the work experiences that they find meaningful is important. We expected that our findings would offer practical insights to support individual PCPs in their careers, encourage others facing challenges, and inform the development of improved specialist training curriculum and more supportive work environments. Rather than generalizing to all physicians working in PC settings, we focused on a specific subgroup — those with demonstrated competencies consistent with PC principles — who are expected to play key roles in Japan’s future healthcare system.

## Methods

### Study design and participants

We employed a qualitative research approach by conducting personal interview surveys [20]. Study participants were PCPs who regularly work in clinics or small hospitals (approximately 100 beds or less). They held either JPCA-certified Family Physician status (obtained by completing a structured training program and passing a specialist board examination) or Diplomate in Primary Care of the JPCA certification (granted based on submitted documentation demonstrating equivalent clinical practice scope). Both certifications formally recognize competencies consistent with PC principles in Japan. Although Diplomates in Primary Care of the JPCA do not complete a standardized training program, the breadth and nature of their clinical practice are considered equivalent to those of JPCA-certified Family Physicians. Of the approximately 50,000 internal medicine physicians working in Japanese clinics as of 2023 [21], about 5,400 are certified as Diplomates in Primary Care and 1,130 hold the full JPCA-certified Family Physician qualification [22]. Although still a minority, these certified PCPs represent a group trained in or committed to the core principles of PCs. Their numbers are expected to grow as Japan’s healthcare system transitions toward a more structured PC model. Accordingly, we intentionally included only certified PCPs to ensure consistency in qualifications and alignment with internationally recognized standards of PC practice. We also selected study participants based on the prevalence of PC in clinics and small hospitals in Japan. Using purposeful sampling [20], we selected physicians meeting these criteria from various regions. This approach minimized bias related to sex, place of residence, institution, and type of work, allowing us to collect diverse information. We explained the purpose of the study to participants via email in advance and obtained their formal written consent on the day of the interview. With reference to previous studies, we planned to interview 10 to 20 participants. This range allowed for conducting additional interviews if needed to ensure that all relevant themes were identified.

### Data collection

Interviews were conducted between October 2021 and February 2022, either in person or via the ZOOM^®^video conference system, using a semi-structured interview guide. The interview guide was developed specifically for this study to explore physicians’ career paths, daily work schedules, perceptions of financial incentives, and experiences which contributed to their sense of meaning and value in their work. Specifically, it aimed to understand the types of work performed in various workplaces and the physicians’ feelings about their current working styles, including workload and aspirations for their professional environment or financial compensation (Supplement material 1). Verbatim transcripts of responses to the interview questions were used as data for analysis. As a pilot study, we conducted a preliminary interview with one participant to confirm the appropriateness of the interview method and questions. The subsequent (first) participant was then interviewed by one of the co-investigators (JH), who has published multiple qualitative research articles. All other participants were interviewed solely by the first author (YY). Face-to-face interviews were conducted in a private room or separate examination room to ensure privacy. ZOOM interviews were conducted with investigators confirming they were alone so as not to be overheard. We scheduled the interviews to take 30 min to 1 h, including an explanation of the study and informed consent. Audio and video recording of the interview began after obtaining consent from the study participant. Potential concerns with online interviews include difficulty establishing rapport, reduced non-verbal communication, and potential distractions or technical disruptions [23, 24]. To mitigate these, the interviewer engaged in informal conversation before beginning the interview and started with easy-to-answer questions to help participants feel comfortable. At the interview’s onset, we collected background data on the participant, including their graduation year, sex, current workplace, and family structure. In the first half of the interview, we asked the study participants about their career histories and current workplaces and tasks. The goal was to encourage them to reflect on and contextualize their professional experiences more deeply. Specifically, the purpose of including this question was to facilitate recall and narrative richness, rather than to conduct comparative analysis based on career stage, and career-stage differences were not analyzed in this study. To facilitate broader recall, we also asked participants about satisfaction with their salary and work environment, and the reasons for their responses. The second half of the interview focused on the question: “What experiences have you had that you feel are meaningful, valuable, or rewarding as a physician’s work?” To maintain neutrality across all participants, we ensured that questions about their career work involvement were asked at the beginning of the interview.

### Data analysis

Data analysis was conducted using inductive thematic analysis, which involved identifying and organizing themes from study participants’ narratives about experiences they found meaningful, valuable, or rewarding as a physician’s work [25]. This method allowed for in-depth exploration of the participants’ responses to the research questions and enabled us to identify patterns and connections between different themes. The specific analysis procedure was as follows. First, we thoroughly read the verbatim transcripts and extracted the relevant segments of text for analysis. Primary codes were then generated from the extracted data. When multiple similar primary codes emerged from different parts of a single participant’s data, they were consolidated into secondary codes. Next, similar primary and/or secondary codes were grouped to form themes. Finally, we reviewed the themes to ensure that they accurately reflected the original data and preserved the meaning of the initial codes. Themes were revised as needed and finalized. As we proceeded with data analysis, we found that even new primary codes extracted from interview data often matched existing secondary codes or themes. Once data analysis for all 14 participants was completed, we confirmed through author discussions that no additional themes emerged, and the analysis was accordingly concluded. The manuscripts were returned to the participants, and member checking was conducted regarding the results of this analysis. There were minor comments regarding the expression of the results, but none that required a change in theme. The main analysis was performed by the first author (YY) and one of the co-investigators (JH) who has experience in qualitative research and thematic analysis. A triangulation of all tasks was performed with another co-investigator (RG), who has extensive experience in qualitative research and is knowledgeable about the current state of PC. YY is a female JPCA-certified family physician who is primarily engaged in outpatient and home visit care at a local clinic, and is also involved in education and research as a university faculty member. JH is a male JPCA-certified family physician and university faculty member who specializes in medical education. He has been involved in part-time outpatient care, home visit care, and emergency care for more than 15 years. RG is a male physiotherapist engaged in research and education as a university faculty member. He is also involved in educating JPCA-certified family physicians and General Medicine fellows of the Japanese Medical Specialty Board.

To address potential biases stemming from prior relationships between interviewers and participants, several strategies were employed. Recognizing the possibility that participants might provide socially desirable responses or tailor answers to perceived expectations [26, 27], the interviews were conducted using a semi-structured format. This allowed for consistent questioning across participants, including asking about topics already known to the interviewer, to ensure uniformity and minimize interviewer influence. Given that familiarity with the participants or industry could lead to confirmation bias, in which the interviewer may unintentionally interpret responses in a manner consistent with their own hypotheses or prior knowledge [28], the analysis focused on the data in a holistic rather than selective fashion. Additionally, member checking was also implemented to verify the accuracy and authenticity of the interpretations [28]. Finally, reflexivity was integrated throughout the research process, which enabled the researchers to critically examine how their prior relationships and positions might have influenced the interviews and subsequent analysis.

This study was conducted with the approval of the Medical Ethics Committee of the Ethics Committee of the University of Tsukuba (approval number 1601), and all participants provided written informed consent before participating.

## Results

### Participant characteristics

One of the 15 study participants was excluded from the study because this participant was not a JPCA-certified family physician or Diplomate in Primary Care of the JPCA. Ultimately, 14 participants agreed to participate in the study, of whom 11 were interviewed by video call. Participant characteristics are shown in Table 1. Nine participants were male, and the mean number of years as a physician was 19.1. Eleven participants were JPCA-certified family physicians and three were Diplomates in Primary Care of the JPCA. Nine participants worked in the Kanto region, and one each in the Hokkaido, Hokuriku, Kinki, Chugoku, and Kyushu regions. The most common main workplace was a clinic, followed by a hospital, university, company, and graduate school. Participants were engaged in a wide range of professional activities, including outpatient care, home visits, inpatient care, nursing home services, community engagement, occupational health, education, and research. Many clinic-based participants also served as directors and participated in organizational management.

**Table 1:** Participant characteristics (*n* = 14)

No	Sex	Place of work	Certification as Primary Care Physician	Types of work facility^*^	Years as a physician	Details of current work
1	male	Kanto region	JPCA-certified Family Physician	university	18	outpatient care, home visit care, education, research, meetings and management
2	male	Kanto region	JPCA-certified Family Physician	clinic	16	outpatient care, home visit care, education, research
3	male	Kanto region	Diplomate in Primary Care of JPCA	clinic	28	outpatient care, home visit care, education, community activities, meetings and management
4	female	Kanto region	JPCA-certified Family Physician	clinic	9	outpatient care, home visit care, education, research
5	male	Hokkaido region	JPCA-certified Family Physician	clinic	17	outpatient care, home visit care, education, community activities, meetings and management
6	male	Kanto region	Diplomate in Primary Care of JPCA	company	26	outpatient care, research, company doctor
7	female	Kyusyu region	JPCA-certified Family Physician	hospital	28	outpatient care, inpatient care, home visit care, education, research, company doctor, meetings and management
8	male	Kanto region	JPCA-certified Family Physician	clinic	13	outpatient care, inpatient care, home visit care, nursing home care, education, community activities, meetings and management
9	male	Kinki region	JPCA-certified Family Physician	hospital	18	outpatient care, inpatient care, home visit cere, emergency care, education, research, meetings and management
10	male	Kanto region	Diplomate in Primary Care of JPCA	clinic	34	outpatient care, home visit care, nursing home care, community activities, meetings and management
11	female	Hokuriku region	JPCA-certified Family Physician	clinic	17	outpatient care, home visit care
12	female	Chugoku region	JPCA-certified Family Physician	clinic	15	home visit care, community activities, education
13	male	Kanto region	JPCA-certified Family Physician	clinic	14	outpatient care, home visit care, community activities, meetings and management
14	female	Kanto region	JPCA-certified Family Physician	graduate student	14	outpatient care, education, research

*JPCA* Japan Primary Care Association

*For participants with multiple workplaces, the facility in which they spent the majority of their working hours was listed

### Work experiences that the primary care physicians found meaningful

A sample of text data, codes, and themes identified regarding work experiences that the participants found meaningful is shown in Supplemental material 2, and a summary of the secondary codes and themes is listed in Table 2. The symbols “ ”, < >, and [] denote a representative sample of text data, codes, and themes, respectively. In addition, the author has added explanations in parentheses () for parts where the meaning was difficult to understand from the context. The analysis identified the following six themes: 1) [Management of diverse health problems], 2) [Comprehensive approach to patients, their families and their issues], 3) [Trust relationships with patients built by continuity], 4) [Experience supporting patients with complex problems through interprofessional collaboration], 5) [Contribution to healthcare provider and medical student education], and 6) [Contribution to the community and society]. Thematic analysis revealed no notable differences in the responses of physicians certified as Diplomates in Primary Care compared to those certified as JPCA-certified PCPs. It was considered that it would not be possible to extract new themes by increasing the number of Diplomates in Primary Care participants. Despite differences in their certification process, both groups shared similar perspectives on meaningful experiences in their practice, suggesting a common foundation in their PC values and clinical engagement. Details on each theme are provided below.

1. [Management of diverse health problems]
  Many of the study participants worked in a medical institution where patients presented with diverse chief complaints or diseases. They felt that the specialty required physicians to < deal[ing] with undifferentiated and diverse symptoms>, such as managing patients with unclear complaints in the early stage of a disease, or accurately diagnosing various conditions, and ensuring the < management of common diseases > in each patient, from initial response to follow-up, for prevalent illnesses. They felt the significance of the routine provision of such care.
  *“I’m interested in many things and want to increase my knowledge of them. As a physician*,* I encounter a myriad of problems with the patient in front of me. In that sense*,* I’m medically motivated all the time*,* for which I value myself as a physician.* (#8)
2. [Comprehensive approach to patients, their families and their issues]
  The study participants found it meaningful to have the < experience of stabilizing complex cases > through a comprehensive biopsychosocial approach, and the < experience of being consulted on both disease- and non-disease-related matters>, including physical problems and life and living concerns. Furthermore, the < experience of providing end-of-life care at home and family care>, facilitated by a holistic understanding of the patient’s medical condition and family context, was considered meaningful. This understanding enabled positive shifts in family dynamics during end-of-life care or the creation of a peaceful environment through family cooperation. The participants also found it meaningful to have the < experience of bridging the gap between departments > by taking a comprehensive view of people in situations where it is not clear which department is most appropriate for providing care (e.g., maternal-neonatal or specialized-palliative care transitions).
  *“For people who frequently come to the emergency department (psychosocial problems and multiple diseases are interrelated)*,* (…) in home visit care*,* I evaluate and manage the patient from every angle by taking a close look at the patient’s lifestyle*,* and that way I can leverage my expertise*,* and this leads to my satisfaction.”* (#9).
  *“It is when we provide good end-of-life care and the family thanks us for it. (…) It makes me feel that I have done a good job when I manage to talk the family into home end-of-life care*,* calming the situation through appropriate communication*,* even when there arises a last minute talk about whether or not to hospitalize the patient in the end-of-life care stage.”* (#13).
3. [Trust relationships with patients built by continuity]
  As the study participants continued to provide medical care, they found meaning in gradually developing a < relationship with patients that allows discussion of any symptoms>, and the experience of becoming a partner to share the patient’s life milestones or the < experience of being relied upon as a valuable presence for patients and their families>, in which they felt trusted by the family as they also examined the patient’s family. They found it meaningful when they had the < experience of being able to provide patients with opportunities to self-reflect on their lifestyles > after a long period of time with no improvement, and realized that they successfully made a positive impact on the patients’ lives.
  *“I have outpatients who I’ve seen for about 15 years now. That makes me feel that we share our lives. (…) I sometimes feel very grateful that they have chosen me as someone with whom they can share (events that occur in their lives).”* (#1).
4. [Experience supporting patients with complex problems through interprofessional collaboration]
  Given that they often encountered complex cases with multiple issues, such as complex family backgrounds and financial problems, in addition to existing illnesses, the study participants found it meaningful to have the < experience of being relied upon and fulfilling their role as a physician through interprofessional collaboration>. They also found meaning in the < experience of promoting smooth information sharing among patients, their families, and multi-professions>, enhancing mutual understanding in various situations (e.g., explanation of patients’ medical conditions to their families, pre-discharge conferences, and conversations with multi-professions in hospital wards). With regard to “multi-professions” here, not all participants specified the details of their occupation. Considering the typical Japanese PC setting, inpatient care involves physicians, nurses, rehabilitation therapists, medical social workers (specialists who support discharge and transfer), and others. In outpatient care and home visit care, physicians, clinic nurses or home visit nurses, home visit rehabilitation therapists, care managers (specialists who coordinate individual patient care), and sometimes administrative staff collaborate according to the patient’s needs. Interprofessional collaboration enabled the study participants to address complex cases often avoided by specialists, exerting their various strengths in a collective fashion based on mutual understanding and thereby fostering the < sense of being able to provide the best care through interprofessional collaboration>.
  *“I find it worthwhile to talk to someone with a different profession and hear them say they want me to take care of that patient. For example*,* there is one public health nurse who often refers patients to me who are in a difficult situation or whose mother has depression*,* although there are a number of clinics or hospitals with a pediatric department. There is also a care manager who*,* for some reason*,* tells other patients to come to see me*,* (…) that’s when I find it meaningful.”* (#5).
  *“We physicians are not alone; we have nurses and rehabilitation physiotherapists playing their roles and administrative staff supporting us. When I realize that we all work together for the patient and his/her family*,* I can feel that we are maximizing the value of the interprofessional team with the power of such a team.”* (#8).
5. [Contribution to healthcare provider and medical student education]
  Most of the study participants were involved in the education of medical students, early-stage physicians-in-training, and PC fellows. In this context, they found meaning in the < experience of being involved in the growth of learners>, and the < experience of serving the community as an educational venue>(e.g., the clinic where they work and their community). The positive meaning of < being involved as a co-learner in lifelong learning > by teaching and learning together with younger physicians was also noted, as was the realization of becoming a < role model as a primary care physician > for those interested in PC.
  *“Lifelong education will not be possible unless students keep coming to us. (…) I use it (explanation about patients and their treatment options) for my lifelong learning. (…) Providing on-the-job training is the best nourishment for me*,* not a commercial effort.”* (#5).
  *“Sometimes*,* students and early-stage physicians-in-training who see me (seeing a patient) say*,* ‘I want to be someone like you who can support patients.’ Frankly*,* it makes me feel happy. It is rewarding to me when I try to show my students that family physicians work with a sense of satisfaction and it is understood by them.”* (#12).
6. [Contribution to the community and society]
  Through engagement in community activities, the study participants found it meaningful to work toward the < building of a team to respond to the needs of the community > and enhancing the team’s performance through strengthened trust relationships for improved comprehensive and integrated medical care in the areas where the participants work. Some participants found meaning in the < experience of being able to respond in a timely manner to the medical needs of their organizations and society > by providing care in a variety of settings, such as responding to outpatient fever needs during the COVID-19 pandemic, serving as school physicians, and conducting local seminars. Participants involved in research found it meaningful to have the < experience of disseminating knowledge that led to solutions to local problems > by studying ongoing issues that they could detect in their medical practice. They also found it meaningful to see such knowledge catch the attention of someone in the world and become useful to society, which made their < experience of having their studies connected to the world > intellectually stimulating.
  *“There is a great social demand for it (establishment of outpatient care for fever related to the COVID-19 pandemic)*,* so I see it (establishment of providing outpatient care for fever) as very valuable.”* (#2).

**Table 2:** Summary of themes and secondary codes

Theme	Secondary code
Management of diverse health problems	‣Dealing with undifferentiated and diverse symptoms
	‣Management of common diseases
Comprehensive approach to patients, their families and their issues	‣Experience of stabilizing complex cases
	‣Experience of being consulted on both disease- and non-disease-related matters
	‣Experience of providing end-of-life care at home and family care
	‣Experience of bridging the gap between departments
Trust relationships with patients built by continuity	‣Relationship with patients that allows discussion of any symptoms
	‣Experience of being relied upon as a valuable presence for patients and their families
	‣Experience of being able to provide patients with opportunities to self-reflect on their lifestyles
Experience supporting patients with complex problems through interprofessional collaboration	‣Experience of being relied upon and fulfilling their role as a physician through interprofessional collaboration
	‣Experience of promoting smooth information sharing among patients, their families, and multi-professions
	‣Sense of being able to provide the best care through interprofessional collaboration
Contribution to healthcare provider and medical student education	‣Experience of being involved in the positive growth of learners
	‣Experience of serving the community as an educational venue
	‣Being involved as a co-learner in lifelong learning
	‣Role model as a primary care physician
Contribution to the community and society	‣Building of a team to respond to the needs of the community
	‣Experience of being able to address the medical needs of their organizations and the society in a timely manner
	‣Experience of disseminating knowledge that led to solutions to local problems
	‣Experience of having their studies connected to the world

### Current salary and work environment

All participants reported satisfaction with their current salary. Regarding the work environment, three noted a heavy workload, but no additional concerns were raised. None of the participants linked these aspects to their meaningful work experiences.

## Discussion

This study identified several experiences that certified PCPs in Japan found meaningful in their work. These experiences were categorized into two key perspectives: micro-level (e.g., relationships with patients and managing diverse cases) and macro-level (e.g., contributions to the community, education, and research). This distinction is consistent with Rosso et al.’s pathways of meaning through agency and communion [13]. Notably, the identified experiences involved both personal skill development and contributions to others, suggesting that meaning in PCPs’ work emerges from a dynamic interaction between individual patients and broader systems.

**Fig. 1:**
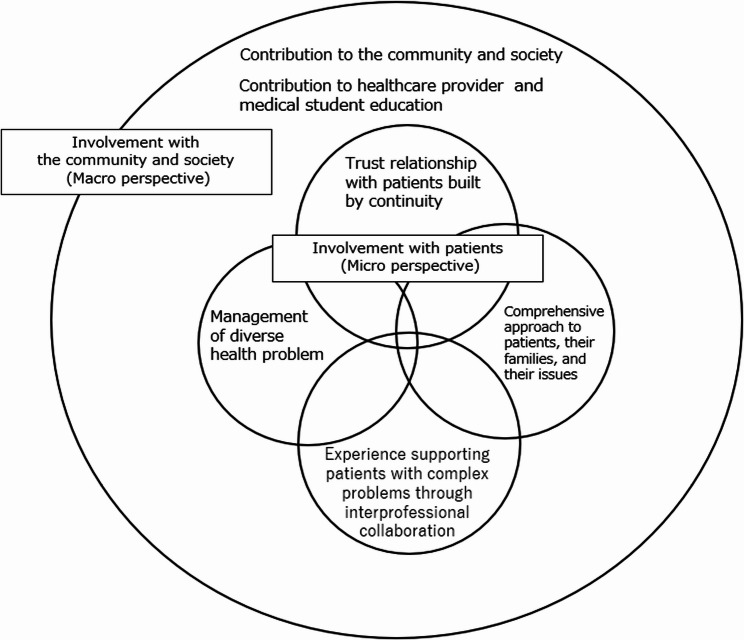
Relationships among identified themes

After completing the thematic analysis, we examined the relationships among the identified themes through iterative discussions. We initially considered the possibility that some micro-level themes, such as “trusting relationships with patients,” might serve as a foundation for others. However, a close examination of the interview data revealed that while trust and continuity were frequently mentioned, they did not exert a clear unidirectional or hierarchical influence on other themes, such as interprofessional collaboration or attention to family context. Instead, these micro-level themes appeared to be mutually interconnected and reinforcing. Therefore, our conceptual model (Fig. 1) represents the micro-level themes as overlapping circles to reflect their interrelated nature rather than a strict hierarchy. Furthermore, we positioned the macro-level themes around the micro-level themes to illustrate the broader social and professional context in which these practices occur.

Clarifying these specific experiences related to the meaning of work may serve to encourage PCPs working in various settings.

Rosso et al. also noted that experiencing multiple meaning-creation pathways strengthens the meaning of work [13]. The themes identified in this study included elements that contribute to others, communities, and society, alongside personal skill enhancement and role fulfillment, suggesting that the meaning of work may be strengthened through various pathways. Prior studies have shown that enhanced meaning in work is positively associated with job satisfaction [15], suggesting that reinforcing such experiences could contribute to sustained engagement and well-being among PCPs.

External factors such as salary and work environment are also known to influence perceptions of meaningful work [13, 29]. In this study, participants reported general satisfaction in these areas, although they did not explicitly connect them to meaningful work. This suggests a tendency for extrinsic factors to provide a supportive foundation, while intrinsic aspects—such as professional autonomy, interpersonal relationships, and opportunities for contribution—appear to play a more central role in shaping what PCPs perceive as meaningful in their work [30]. A point that warrants attention is that, although participants expressed satisfaction with their salary, this may reflect cultural or psychological discomfort in openly discussing financial concerns, rather than an absence of discontent.

Previous research has identified clinical stressors like difficulty resolving patient problems, systemic issues such as low income and long hours, and a lack of understanding from specialists [31–33]. However, studies show that individuals can find meaning in their work even amidst challenging circumstances [34]. This sense of meaning enhances resilience, aids in problem-solving, and mitigates the negative effects of work stress on their personal lives [35, 36] Therefore, by actively addressing these challenges where possible and finding meaning in their work, PCPs can potentially sustain their careers without losing their professional identity and may achieve a more fulfilling professional life.

On comparison of the results of this survey with the principles and definitions of family medicine/general practice around the world, many of the codes and themes identified in this study were shown to be derived from the five principles of PC: accessibility, comprehensiveness, coordination, continuity, and accountability [37]. This could reflect the influence of education in the training of PCPs in Japan, implying that the principles of PCPs are generally well-established. When we compared the themes identified in this study with the WONCA Europe definition of general practice/family medicine [38], we observed substantial overlap, particularly in areas such as comprehensive clinical care, continuity, and community orientation. WONCA Europe also defines one theme of the specialty of PCPs as follows: “They do this by caring for the ecosystem made up of people, animals, and the natural environment, being aware of their unique position as role models for their patients regarding the promotion of leading a sustainable way of life.” However, our participants did not mention such planetary health or environmental responsibility as meaningful components of their work. The concept of planetary health was first proposed in 2015 [39], but in Japan, the term “planetary health” has only begun to be recognized in recent years. It was not until the 2020 s that academic organizations began making policy recommendations and promoting awareness campaigns and research [40, 41]. While the concept is gaining understanding in the context of recent extreme weather events, it has not yet been integrated into medical education or daily clinical practice among certified PCPs in Japan. Conversely, contributions to medical education and research, which were highlighted by many participants, are not explicitly stated in the WONCA definition, but may be considered essential in the Japanese context where certified PCPs often serve as educators and mentors. These differences suggest that while core values are shared globally, the interpretation and emphasis of “meaningful work” are influenced by local roles and expectations. While the themes identified in this study may resemble the core values and principles of PC, they were derived from real experiences rather than idealized expectations. Importantly, not all participants expressed personal experience with every theme. Their reflections were influenced by factors such as career history, current work setting, and professional responsibilities. These variations highlight the contextual nature of meaningful work and support a more nuanced, practically grounded understanding of how PCPs maintain purpose across diverse settings and career stages.

These findings have practical implications for the support of PCPs. For individuals struggling with their careers, sharing these identified meaningful experiences can provide personal encouragement and a renewed sense of purpose. Furthermore, for supervisors and program directors, these insights can provide valuable hints for designing better specialist training curricula and fostering a more supportive work environment. Specifically, emphasizing opportunities for continuous and comprehensive care, integrated practice and teaching through co-learning, and active community engagement could enhance PCPs’ motivation, resilience, and long-term retention.

Although this study did not aim to assess educational programs, the emphasis placed by participants on co-learning, mentorship, and the integration of practice and teaching suggests that such experiences could inform the design of future medical education curricula that aim to foster professional identity and engagement in PC. Furthermore, while this study focused on the personal experience of meaningful work, the themes identified may also inform broader system-level strategies. For example, ensuring opportunities for continuity of care, supporting educational roles, and promoting community engagement may help sustain motivation and resilience among PCPs. Therefore, future research may further investigate how such elements relate to job satisfaction and long-term retention in PC.

With regard to the study methodology, online interviews are sometimes considered less effective in terms of rapport building and communication depth. However, we found no substantial difference in the richness or content of the responses, likely attributable to the participants’ familiarity with online platforms gained during the COVID-19 pandemic, and to our efforts to create a comfortable environment through pre-interview conversation and a thoughtfully designed interview guide. We therefore consider that the use of online interviews did not significantly affect the quality of the data collected in this study.

These findings may offer insights of relevance to PCPs in countries with similar demographic or healthcare trends and could contribute, albeit indirectly, to broader discussions on the development of PC systems worldwide.

### Limitations

One limitation of this study was a slight imbalance in participant characteristics, such as workplace, which potentially introduced a degree of response bias. For example, there were nine participants working at clinics, compared to two at hospitals, and nine participants living in the Kanto region, accounting for the majority. Furthermore, the significant variation in meaningful experiences based on educational background, workplace, and personal values suggested potential unidentified experiences. However, the inclusion of PCPs in healthcare service, education, and/or research was aimed to avoid bias, and the absence of further themes in the analysis suggested that a certain level of completeness has been ensured. Second, this study did not collect data on participant age, which may have provided additional insights into generational perspectives on meaningful work. Future studies should consider including age and other demographic variables to deepen the analysis. Third, since the interviewer and some of the participants were already acquainted, there is a possibility that the answers were biased toward what the interviewer or society would consider desirable. When analyzing the data, we considered whether this relationship might have influenced the interviews, but since the answers mainly concerned specific personal experiences, we determined that it did not have a significant influence on the results. Fourth, as with all qualitative studies, the interpretation of themes involves a degree of subjectivity, which may influence how findings are derived and presented. Although we employed a predefined and systematic coding process and involved multiple researchers to enhance credibility, complete objectivity cannot be ensured. Additionally, the findings are context-specific and may not be generalizable to all PC settings or healthcare systems. Finally, the study population was limited to certified PCPs — those formally trained or assessed based on comprehensive primary care competencies. As such, the findings may not fully represent the broader population of physicians providing PC in Japan, particularly those with organ-based or non-certified backgrounds. However, given their increasing institutional and societal roles, the perspectives of certified PCPs provide valuable insights into the evolving identity and practice of PC in Japan. Even with these limitations in mind, our revelation of specific experiences of PCPs connected to the meaning of their work may inspire PCPs working in a variety of settings.

## Conclusion

This study found that certified PCPs derive meaning from their work through clinical expertise, long-term patient care, and engagement in community and educational activities — factors which notably embody the core principles of PC.

## Supplementary Information

Supplementary Material 1.

Supplementary Material 2.

## Data Availability

Data is provided within the manuscript or supplementary information file.

## Abbreviations

PC: Primary care
PCP: Primary care physician
JPCA: Japan Primary Care Association
WONCA: World Organization of National Colleges, Academies and Academic Associations of General Practitioners/Family Physicians
COVID-19: Coronavirus infectious disease, emerged in 2019

## References

[1] WONCA in brief. https://www.globalfamilydoctor.com/aboutwonca/brief.aspx. Accessed 31 Jan 2025.

[2] In: Kringos DS, Boerma WGW, Hutchinson A, Saltman RB, editors. Building primary care in a changing Europe. Copenhagen: World Health Organization; 2015. 29035488

[3] Mihara T, Okada T, Fujinuma Y et al. International Comparison of General Practice. Report on Research on the effects of general practice on specialists and interprofessional collaboration in community healthcare. 2018.

[4] Takamura A. The present circumstance of primary care in Japan. Qual Prim Care. 2015;23(5):262–6.

[5] Katori T. Japan’s healthcare delivery system: from its historical evolution to the challenges of a super-aged society. Glob Health Med. 2024;6(1):6–12. 38450110 10.35772/ghm.2023.01121 PMC10912799

[6] Nojiri S, Itoh H, Kasai T, et al. Comorbidity status in hospitalized elderly in japan: analysis from National database of health insurance claims and specific health checkups. Sci Rep. 2019;9(1):20237. 31882961 10.1038/s41598-019-56534-4 PMC6934653

[7] Kato D, Ryu H, Matsumoto T, et al. Building primary care in japan: literature review. J Gen Fam Med. 2019;20(5):170–9. 31516802 10.1002/jgf2.252 PMC6732569

[8] History of JPCA. https://www.primarycare-japan.com/assoc/english/en-history/. Accessed 31 Jan 2025.

[9] Ozone S, Kimura T, Ito M. Survey on the Scope of Practice of General Practitioners. Report on Research on the effects of general practice on specialists and interprofessional collaboration in community healthcare. 2018.

[10] Matsui Y. History and current status of primary care, family medicine, and general practice in Japan and the world. In: Japan Primary Care Association, editor. Japan Primary Care Association Basic Training Handbook. 2021:10–7.

[11] Toi K, Yoshimoto H, Nishigori H. Career planning for young Generalists — What path should they take after becoming a certified family physician?? Official J Jpn Prim Care Assoc. 2014;37(4):374–6.

[12] Ie K, Tahara M, Murata A, et al. Factors associated to the career choice of family medicine among Japanese physicians: the dawn of a new era. Asia Pac Fam Med. 2015;3(4):185–90. 10.1186/s12930-014-0011-2 PMC5377022 28392748

[13] Rosso BD, Dekas KH, Wrzesniewski A. On the meaning of work: A theoretical integration and review. Res Organ Behav. 2010;30:91–127.

[14] Steger MF, Dik BJ, Duffy RD. Measuring meaningful work. J Career Assess. 2012;20:322–37.

[15] Allan BA, Batz-Barbarich C, Sterling HM, et al. Outcomes of meaningful work: A Meta-Analysis. J Manag Stud. 2018;56(3):500–28.

[16] Halvorsen PA, Edwards A, Aaraas IJ, et al. What professional activities do general practitioners find most meaningful? Cross sectional survey of Norwegian general practitioners. BMC Fam Pract. 2013;14:41. 23522393 10.1186/1471-2296-14-41 PMC3615944

[17] Al-Ghamdi S, Alajmi M, Batais MA, et al. Meaningful professional activities from family medicine practitioners’ perspectives: a study from Saudi Arabia. Prim Health Care Res Dev. 2021;22(e13):1–6. 10.1017/S1463423621000104 PMC8101074 33818367

[18] Yamamoto Y, Haruta J, Goto R, et al. What kinds of work do Japanese primary care physicians who derive greater positive meaning from work engage in? A cross-sectional study. J Gen Fam Med. 2023;24(2):94–101. 36909785 10.1002/jgf2.595 PMC10000278

[19] Saif-Ur-Rahman KM, Onishi J, Mamun R, et al. Y. Job satisfaction among physicians providing health care to the elderly in japan: a qualitative study. Psychogeriatrics. 2021;21(3):311–6. 33598980 10.1111/psyg.12668

[20] Dicicco-Bloom B, Crabtree BF. The qualitative research interview. Med Educ. 2006;40(4):314–21. 16573666 10.1111/j.1365-2929.2006.02418.x

[21] Overview of Statistics on Physicians, Dentists, and Pharmacists. chrome-extension://efaidnbmnnnibpcajpcglclefindmkaj/https://www.mhlw.go.jp/toukei/saikin/hw/ishi/22/dl/R04_kekka-1.pdf. Accessed 1 May 2025.

[22] Increasing number of Doctors switching from other fields to general practice- - Interview with Tessyu kusaba, president of the Japan primary care association ◆vol.4 https://www.m3.com/news/iryoishin/1128734

[23] Irani E. The use of videoconferencing for qualitative interviewing: opportunities, challenges, and considerations. Clin Nurs Res. 2019;28(1):3–8. 30470151 10.1177/1054773818803170

[24] Engward H, Goldspink S, Iancu M, Kersey T, Wood A. Togetherness in separation: practical considerations for doing remote qualitative interviews ethically. Int J Qual Methods. 2022;21:1–9.

[25] Nowell LS, Norris JM, White DE et al. Thematic analysis: Striving to Meet the Trustworthiness Criteria. Int J Qual Methods. 2017;16(1). 10.1177/1609406917733847.

[26] Alam MK. Does the relationship between the interviewer and interviewee matter in qualitative research? ICRRD J. 2024;5(1):140–5.

[27] Horsfall M, Eikelenboom M, Draisma S, et al. The effect of rapport on data quality in Face-to-Face interviews: beneficial or detrimental?? Int J Environ Res Public Health. 2021;18:10858. 34682600 10.3390/ijerph182010858 PMC8535677

[28] Mehra B. Bias in qualitative research: voices from an online classroom. Qualitative Rep. 2002;7(1):1–19.

[29] Beraldo KA, Mauricio NMM, de Lima TC, de Bezerra N. Rodrigues TA Das NM. The meanings of work: a study in the perception of nurses. Rev Obs. 2020;6:a12.

[30] Nikolova M, Cnossen F. What makes work meaningful and why economists should care about it. Labour Econ. 2020;65:101847. 10.1016/j.labeco.2020.101847.

[31] Fairhurst K, May C. What general practitioners find satisfying in their work: implications for health care system reform. Ann Fam Med. 2006;4(6):500–5. 17148627 10.1370/afm.565 PMC1687165

[32] Ham IV, Verhoeven AAH, Groenier KH, et al. Job satisfaction among general practitioners: A systematic literature review. Eur J Gen Pract. 2009;12:174–80. 10.1080/13814780600994376 17127604

[33] Manca DP, Varnhagen S, Brett-MacLean P, et al. Rewards and challenges of family practice Web-based survey using the Delphi method. Can Fam Physician. 2007;53:277–86. 17872645 PMC1949127

[34] Stephenson AL, Bell N. Finding meaningful work in difficult circumstances: A study of prison healthcare workers. Health Serv Manage Res. 2019;32(2):69–77. 29999425 10.1177/0951484818787698

[35] Allan BA, Douglass RP, Duffy RD, et al. Meaningful work as a moderator of the relation between work stress and meaning in life. J Career Assess. 2016;24(3):429–40.

[36] Kanwal A, Maqsood R, Karim M. The impact of meaningful work on employee identity and the mediating role of employee resilience. J Appl Res Multidisciplinary Stud. 2020;1(1):30–47.

[37] Institute of Medicine. Chapter 2 Primary health care difined. In: A Manpower Policy for Primary Health Care: Report of a Study. 10.17226/9932. Accessed 31 Jan 2025.

[38] The European Definition of GP/FM. Chrome-extension://efaidnbmnnnibpcajpcglclefindmkaj/ https://www.woncaeurope.org/file/41f61fb9-47d5-4721-884e-603f4afa6588/WONCA_European_Definitions_2_v7.pdf. Accessed 1 May 2025.

[39] Horton R, Lob S. Planetary health: a new science for exceptional action. Lancet. 2015;386(10007):1921–22. 26188746 10.1016/S0140-6736(15)61038-8

[40] About Planetary Health. https://hgpi.org/en/tag/planetary-health. Accessed 25 July 2025.

[41] Climate Emergency Declaration in Primary. Care ~ Primary Care for a Healthy Planet ~ chrome-extension://efaidnbmnnnibpcajpcglclefindmkaj/https://www.primarycare-japan.com/files/news/news-834-1.pdf. Accessed 25 July 2025.

