# Supplementary material for: Meaningful work experiences of certified primary care physicians in Japan: a qualitative study
Source: BMC Prim Care. 2025 Oct 28;26:328. doi: 10.1186/s12875-025-03026-2 (PMC12560313; doi:10.1186/s12875-025-03026-2)
Supplement: Supplementary file 1 — Supplementary Material 1. [file 12875_2025_3026_MOESM1_ESM.docx]

**Semi-structured interview questionnaire**

Date:

Research No.:

Interviewer:

□ Explain the research to the research subject and have them read the consent form.

□ Have them fill in the consent form, and then send it to us by email later.

Questionnaire items

□ Sex

□ Year of graduation

□ Current workplace

□ Family structure (living alone or together with others)

□ First of all, please tell me about your career to date. Specifically, what kind of work have you done at what kind of workplace?

□ Next, please tell me about your current daily work schedule (what do you do on which days; how many times a week or month do you work overtime, night shift, or on call at home; etc.) How do you feel about your current working style? (for example, whether the workload is too much or just right; and any wishes you have regarding other professions or the work environment, financial incentives such as salary, etc.)

□ (If financial incentives are not mentioned) Do you think that the financial reward for your work is appropriate for the work you do and the quality of your work? (If you don't mind,) Please tell us why.

□What experiences have you had that you feel are meaningful, valuable, or rewarding in terms of a physician’s work? Please include experiences outside of working hours.

Ask “Are there any others?” several times.

Insert a summary in the middle.

□ How did these experiences lead to you value and find meaning in the work of a physician?

□ When did you start to feel that the work you just talked about was meaningful?

□ When you were a medical student or a junior resident, what was your image of primary care?

□ How does your work as a primary care physician compare to your image of it?

□ What do you think are the issues facing primary care physicians in Japan?

□ Is there anything you forgot to mention?
