# Supplementary material for: Meaningful work experiences of certified primary care physicians in Japan: a qualitative study
Source: BMC Prim Care. 2025 Oct 28;26:328. doi: 10.1186/s12875-025-03026-2 (PMC12560313; doi:10.1186/s12875-025-03026-2)
Supplement: Supplementary file 2 — Supplementary Material 2. [file 12875_2025_3026_MOESM2_ESM.pptx]

## Slide 1
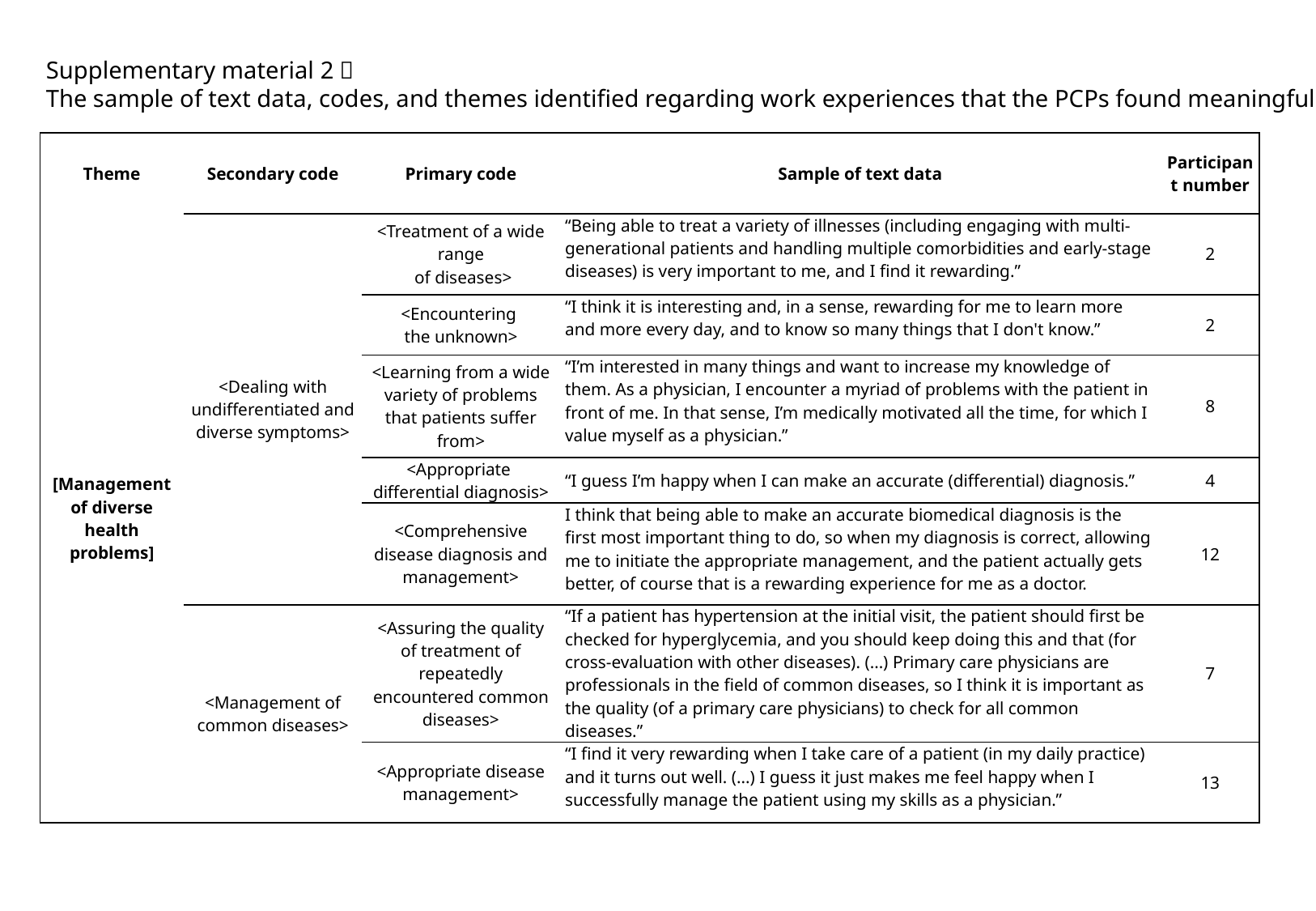

Supplementary material 2：
The sample of text data, codes, and themes identified regarding work experiences that the PCPs found meaningful
| Theme | Secondary code | Primary code | Sample of text data | Participant number |
| --- | --- | --- | --- | --- |
| [Management of diverse health problems] | <Dealing with undifferentiated and diverse symptoms> | <Treatment of a wide range of diseases> | “Being able to treat a variety of illnesses (including engaging with multi-generational patients and handling multiple comorbidities and early-stage diseases) is very important to me, and I find it rewarding.” | 2 |
| | | <Encountering the unknown> | “I think it is interesting and, in a sense, rewarding for me to learn more and more every day, and to know so many things that I don't know.” | 2 |
| | | <Learning from a wide variety of problems that patients suffer from> | “I’m interested in many things and want to increase my knowledge of them. As a physician, I encounter a myriad of problems with the patient in front of me. In that sense, I’m medically motivated all the time, for which I value myself as a physician.” | 8 |
| | | <Appropriate differential diagnosis> | “I guess I’m happy when I can make an accurate (differential) diagnosis.” | 4 |
| | | <Comprehensive disease diagnosis and management> | I think that being able to make an accurate biomedical diagnosis is the first most important thing to do, so when my diagnosis is correct, allowing me to initiate the appropriate management, and the patient actually gets better, of course that is a rewarding experience for me as a doctor. | 12 |
| | <Management of common diseases> | <Assuring the quality of treatment of repeatedly encountered common diseases> | “If a patient has hypertension at the initial visit, the patient should first be checked for hyperglycemia, and you should keep doing this and that (for cross-evaluation with other diseases). (...) Primary care physicians are professionals in the field of common diseases, so I think it is important as the quality (of a primary care physicians) to check for all common diseases.” | 7 |
| | | <Appropriate disease management> | “I find it very rewarding when I take care of a patient (in my daily practice) and it turns out well. (...) I guess it just makes me feel happy when I successfully manage the patient using my skills as a physician.” | 13 |

## Slide 2
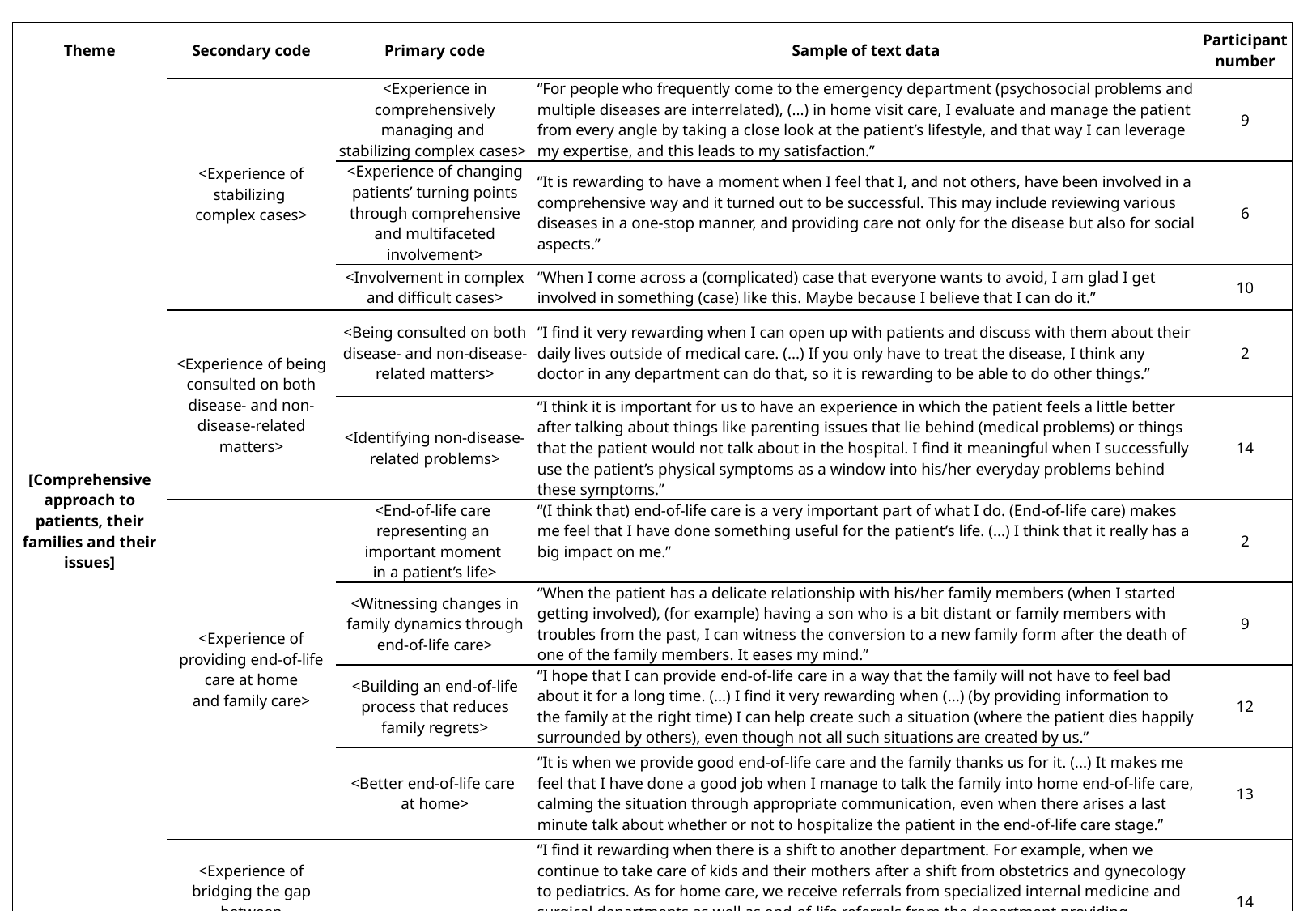

| Theme | Secondary code | Primary code | Sample of text data | Participant number |
| --- | --- | --- | --- | --- |
| [Comprehensive approach to patients, their families and their issues] | <Experience of stabilizing complex cases> | <Experience in comprehensively managing and stabilizing complex cases> | “For people who frequently come to the emergency department (psychosocial problems and multiple diseases are interrelated), (...) in home visit care, I evaluate and manage the patient from every angle by taking a close look at the patient’s lifestyle, and that way I can leverage my expertise, and this leads to my satisfaction.” | 9 |
| | | <Experience of changing patients’ turning points through comprehensive and multifaceted involvement> | “It is rewarding to have a moment when I feel that I, and not others, have been involved in a comprehensive way and it turned out to be successful. This may include reviewing various diseases in a one-stop manner, and providing care not only for the disease but also for social aspects.” | 6 |
| | | <Involvement in complex and difficult cases> | “When I come across a (complicated) case that everyone wants to avoid, I am glad I get involved in something (case) like this. Maybe because I believe that I can do it.” | 10 |
| | <Experience of being consulted on both disease- and non-disease-related matters> | <Being consulted on both disease- and non-disease-related matters> | “I find it very rewarding when I can open up with patients and discuss with them about their daily lives outside of medical care. (...) If you only have to treat the disease, I think any doctor in any department can do that, so it is rewarding to be able to do other things.” | 2 |
| | | <Identifying non-disease-related problems> | “I think it is important for us to have an experience in which the patient feels a little better after talking about things like parenting issues that lie behind (medical problems) or things that the patient would not talk about in the hospital. I find it meaningful when I successfully use the patient’s physical symptoms as a window into his/her everyday problems behind these symptoms.” | 14 |
| | <Experience of providing end-of-life care at home and family care> | <End-of-life care representing an important moment in a patient’s life> | “(I think that) end-of-life care is a very important part of what I do. (End-of-life care) makes me feel that I have done something useful for the patient’s life. (...) I think that it really has a big impact on me.” | 2 |
| | | <Witnessing changes in family dynamics through end-of-life care> | “When the patient has a delicate relationship with his/her family members (when I started getting involved), (for example) having a son who is a bit distant or family members with troubles from the past, I can witness the conversion to a new family form after the death of one of the family members. It eases my mind.” | 9 |
| | | <Building an end-of-life process that reduces family regrets> | “I hope that I can provide end-of-life care in a way that the family will not have to feel bad about it for a long time. (...) I find it very rewarding when (...) (by providing information to the family at the right time) I can help create such a situation (where the patient dies happily surrounded by others), even though not all such situations are created by us.” | 12 |
| | | <Better end-of-life care at home> | “It is when we provide good end-of-life care and the family thanks us for it. (...) It makes me feel that I have done a good job when I manage to talk the family into home end-of-life care, calming the situation through appropriate communication, even when there arises a last minute talk about whether or not to hospitalize the patient in the end-of-life care stage.” | 13 |
| | <Experience of bridging the gap between departments> | | “I find it rewarding when there is a shift to another department. For example, when we continue to take care of kids and their mothers after a shift from obstetrics and gynecology to pediatrics. As for home care, we receive referrals from specialized internal medicine and surgical departments as well as end-of-life referrals from the department providing treatment of cancer for patients. As a family physician, I find it most meaningful to be able to bridge the gaps between these departments.” | 14 |

## Slide 3
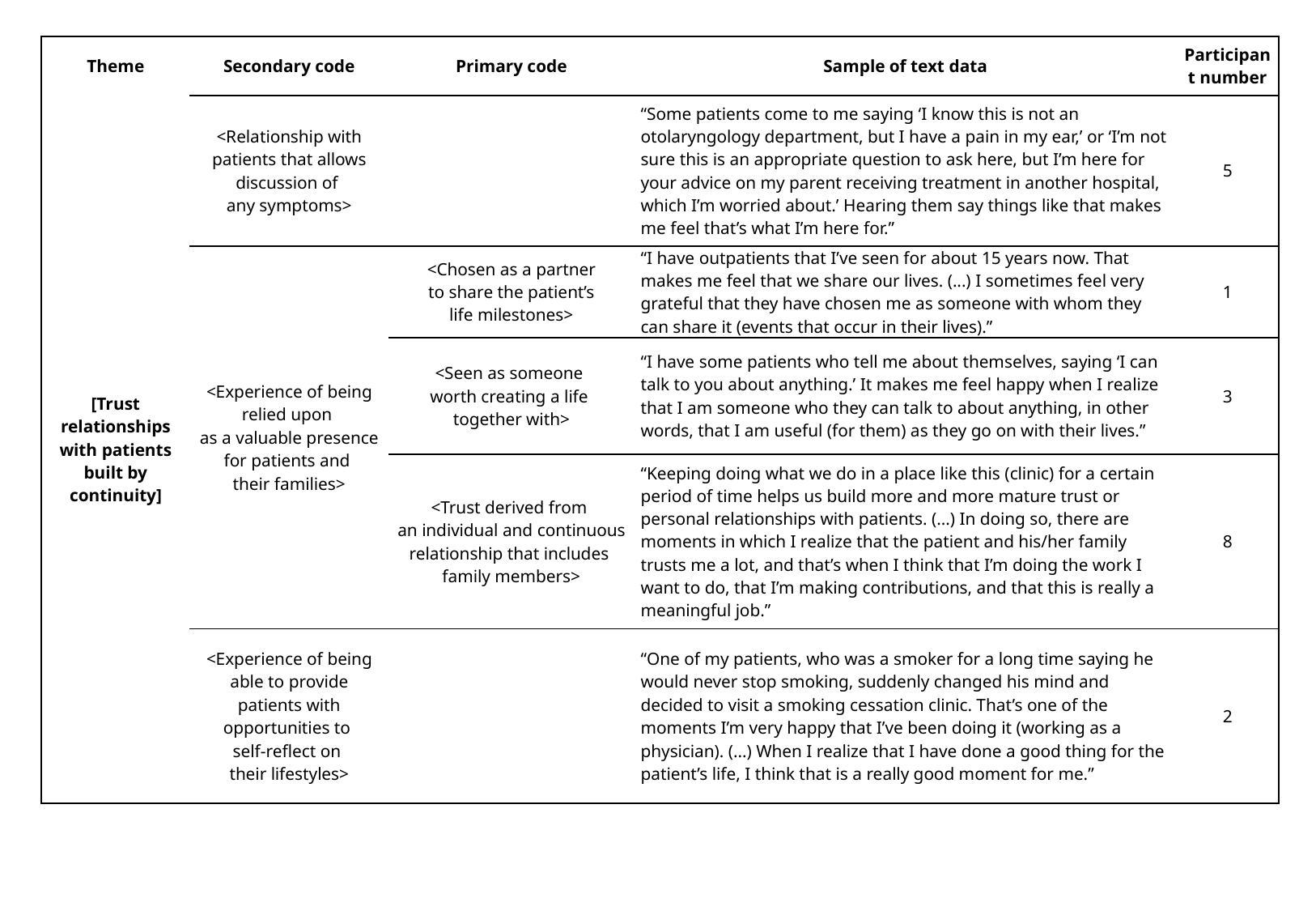

| Theme | Secondary code | Primary code | Sample of text data | Participant number |
| --- | --- | --- | --- | --- |
| [Trust relationships with patients built by continuity] | <Relationship with patients that allows discussion of any symptoms> | | “Some patients come to me saying ‘I know this is not an otolaryngology department, but I have a pain in my ear,’ or ‘I’m not sure this is an appropriate question to ask here, but I’m here for your advice on my parent receiving treatment in another hospital, which I’m worried about.’ Hearing them say things like that makes me feel that’s what I’m here for.” | 5 |
| | <Experience of being relied upon as a valuable presence for patients and their families> | <Chosen as a partner to share the patient’s life milestones> | “I have outpatients that I’ve seen for about 15 years now. That makes me feel that we share our lives. (...) I sometimes feel very grateful that they have chosen me as someone with whom they can share it (events that occur in their lives).” | 1 |
| | | <Seen as someone worth creating a life together with> | “I have some patients who tell me about themselves, saying ‘I can talk to you about anything.’ It makes me feel happy when I realize that I am someone who they can talk to about anything, in other words, that I am useful (for them) as they go on with their lives.” | 3 |
| | | <Trust derived from an individual and continuous relationship that includes family members> | “Keeping doing what we do in a place like this (clinic) for a certain period of time helps us build more and more mature trust or personal relationships with patients. (...) In doing so, there are moments in which I realize that the patient and his/her family trusts me a lot, and that’s when I think that I’m doing the work I want to do, that I’m making contributions, and that this is really a meaningful job.” | 8 |
| | <Experience of being able to provide patients with opportunities to self-reflect on their lifestyles> | | “One of my patients, who was a smoker for a long time saying he would never stop smoking, suddenly changed his mind and decided to visit a smoking cessation clinic. That’s one of the moments I’m very happy that I’ve been doing it (working as a physician). (...) When I realize that I have done a good thing for the patient’s life, I think that is a really good moment for me.” | 2 |

## Slide 4
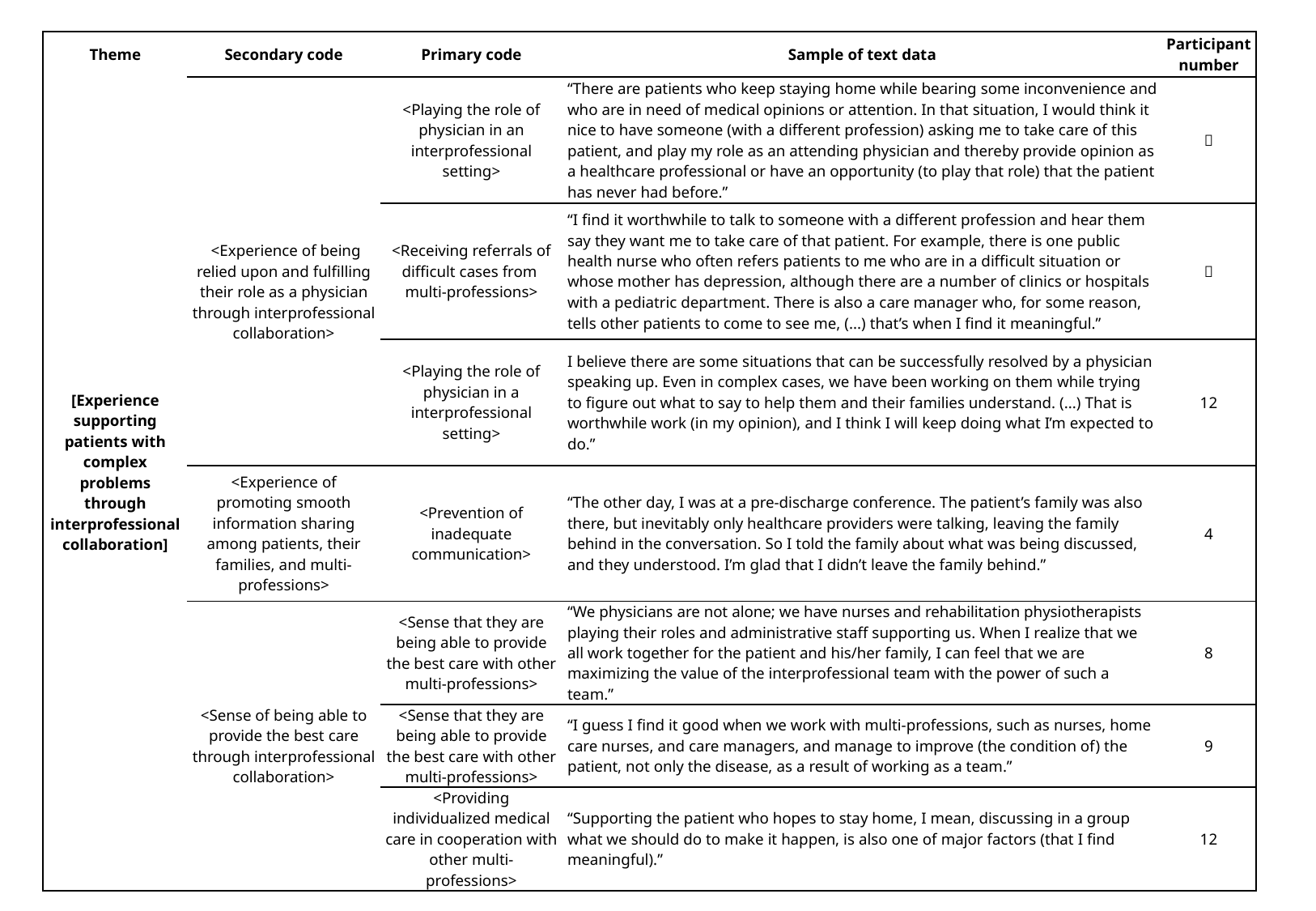

| Theme | Secondary code | Primary code | Sample of text data | Participant number |
| --- | --- | --- | --- | --- |
| [Experience supporting patients with complex problems through interprofessional collaboration] | <Experience of being relied upon and fulfilling their role as a physician through interprofessional collaboration> | <Playing the role of physician in an interprofessional setting> | “There are patients who keep staying home while bearing some inconvenience and who are in need of medical opinions or attention. In that situation, I would think it nice to have someone (with a different profession) asking me to take care of this patient, and play my role as an attending physician and thereby provide opinion as a healthcare professional or have an opportunity (to play that role) that the patient has never had before.” | ３ |
| | | <Receiving referrals of difficult cases from multi-professions> | “I find it worthwhile to talk to someone with a different profession and hear them say they want me to take care of that patient. For example, there is one public health nurse who often refers patients to me who are in a difficult situation or whose mother has depression, although there are a number of clinics or hospitals with a pediatric department. There is also a care manager who, for some reason, tells other patients to come to see me, (...) that’s when I find it meaningful.” | ５ |
| | | <Playing the role of physician in a interprofessional setting> | I believe there are some situations that can be successfully resolved by a physician speaking up. Even in complex cases, we have been working on them while trying to figure out what to say to help them and their families understand. (...) That is worthwhile work (in my opinion), and I think I will keep doing what I’m expected to do.” | 12 |
| | <Experience of promoting smooth information sharing among patients, their families, and multi-professions> | <Prevention of inadequate communication> | “The other day, I was at a pre-discharge conference. The patient’s family was also there, but inevitably only healthcare providers were talking, leaving the family behind in the conversation. So I told the family about what was being discussed, and they understood. I’m glad that I didn’t leave the family behind.” | 4 |
| | <Sense of being able to provide the best care through interprofessional collaboration> | <Sense that they are being able to provide the best care with other multi-professions> | “We physicians are not alone; we have nurses and rehabilitation physiotherapists playing their roles and administrative staff supporting us. When I realize that we all work together for the patient and his/her family, I can feel that we are maximizing the value of the interprofessional team with the power of such a team.” | 8 |
| | | <Sense that they are being able to provide the best care with other multi-professions> | “I guess I find it good when we work with multi-professions, such as nurses, home care nurses, and care managers, and manage to improve (the condition of) the patient, not only the disease, as a result of working as a team.” | 9 |
| | | <Providing individualized medical care in cooperation with other multi-professions> | “Supporting the patient who hopes to stay home, I mean, discussing in a group what we should do to make it happen, is also one of major factors (that I find meaningful).” | 12 |

## Slide 5
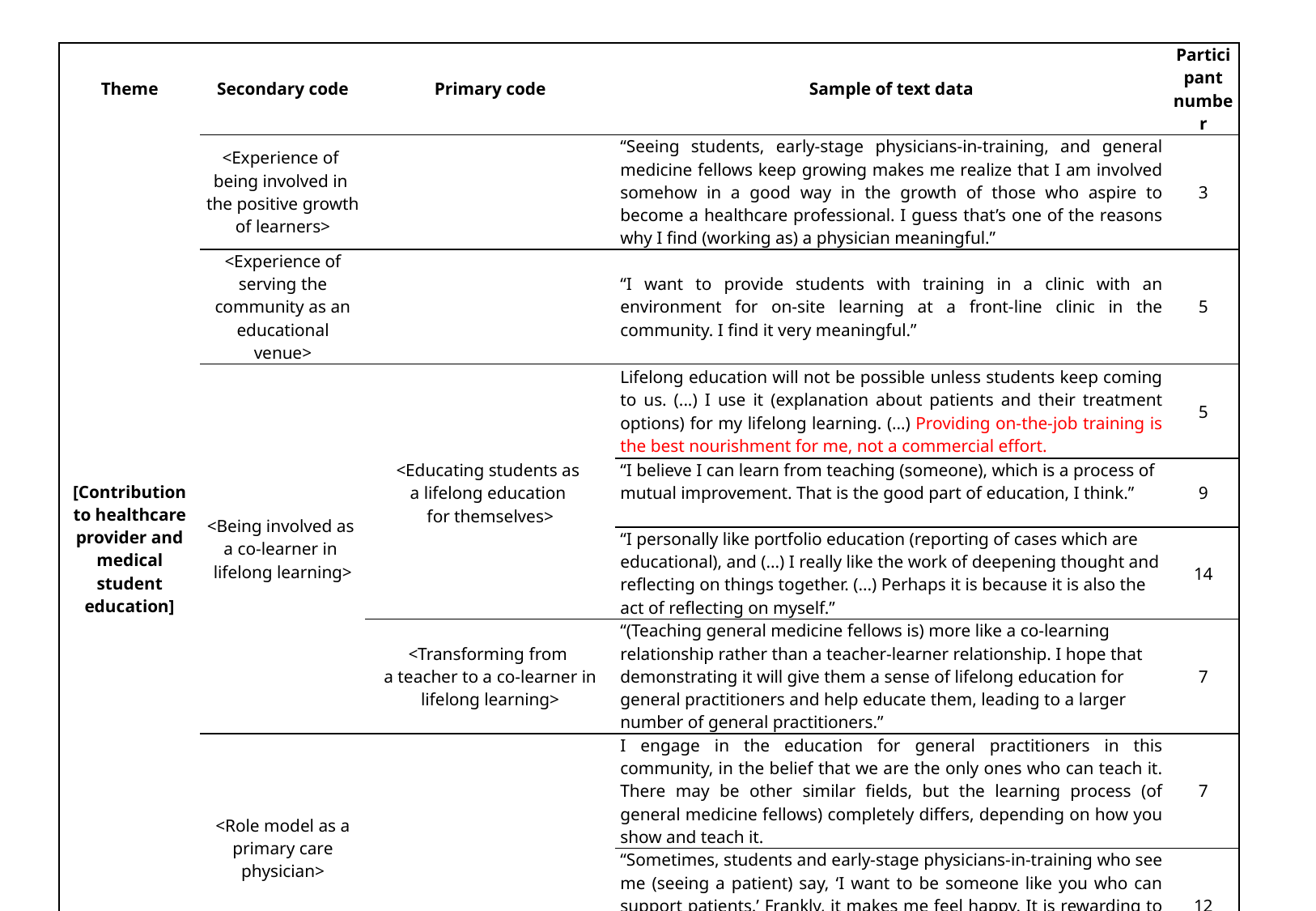

| Theme | Secondary code | Primary code | Sample of text data | Participant number |
| --- | --- | --- | --- | --- |
| [Contribution to healthcare provider and medical student education] | <Experience of being involved in the positive growth of learners> | | “Seeing students, early-stage physicians-in-training, and general medicine fellows keep growing makes me realize that I am involved somehow in a good way in the growth of those who aspire to become a healthcare professional. I guess that’s one of the reasons why I find (working as) a physician meaningful.” | 3 |
| | <Experience of serving the community as an educational venue> | | “I want to provide students with training in a clinic with an environment for on-site learning at a front-line clinic in the community. I find it very meaningful.” | 5 |
| | <Being involved as a co-learner in lifelong learning> | | Lifelong education will not be possible unless students keep coming to us. (...) I use it (explanation about patients and their treatment options) for my lifelong learning. (...) Providing on-the-job training is the best nourishment for me, not a commercial effort. | 5 |
| | | <Educating students as a lifelong education for themselves> | “I believe I can learn from teaching (someone), which is a process of mutual improvement. That is the good part of education, I think.” | 9 |
| | | | “I personally like portfolio education (reporting of cases which are educational), and (...) I really like the work of deepening thought and reflecting on things together. (...) Perhaps it is because it is also the act of reflecting on myself.” | 14 |
| | | <Transforming from a teacher to a co-learner in lifelong learning> | “(Teaching general medicine fellows is) more like a co-learning relationship rather than a teacher-learner relationship. I hope that demonstrating it will give them a sense of lifelong education for general practitioners and help educate them, leading to a larger number of general practitioners.” | 7 |
| | <Role model as a primary care physician> | | I engage in the education for general practitioners in this community, in the belief that we are the only ones who can teach it. There may be other similar fields, but the learning process (of general medicine fellows) completely differs, depending on how you show and teach it. | 7 |
| | | | “Sometimes, students and early-stage physicians-in-training who see me (seeing a patient) say, ‘I want to be someone like you who can support patients.’ Frankly, it makes me feel happy. It is rewarding to me when I try to show my students that family physicians work with a sense of satisfaction and it is understood by them.” | 12 |

## Slide 6
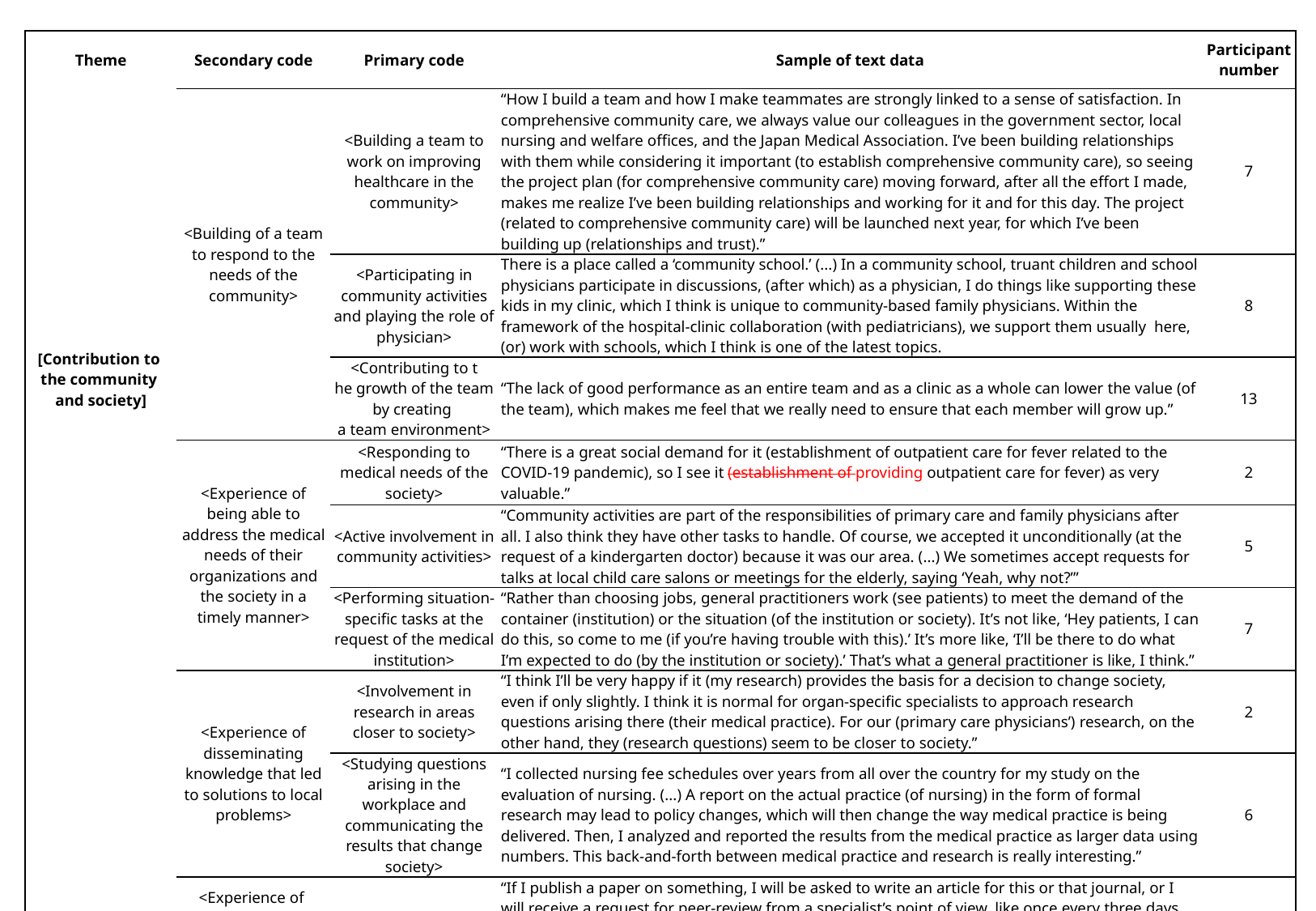

| Theme | Secondary code | Primary code | Sample of text data | Participant number |
| --- | --- | --- | --- | --- |
| [Contribution to the community and society] | <Building of a team to respond to the needs of the community> | <Building a team to work on improving healthcare in the community> | “How I build a team and how I make teammates are strongly linked to a sense of satisfaction. In comprehensive community care, we always value our colleagues in the government sector, local nursing and welfare offices, and the Japan Medical Association. I’ve been building relationships with them while considering it important (to establish comprehensive community care), so seeing the project plan (for comprehensive community care) moving forward, after all the effort I made, makes me realize I’ve been building relationships and working for it and for this day. The project (related to comprehensive community care) will be launched next year, for which I’ve been building up (relationships and trust).” | 7 |
| | | <Participating in community activities and playing the role of physician> | There is a place called a ‘community school.’ (...) In a community school, truant children and school physicians participate in discussions, (after which) as a physician, I do things like supporting these kids in my clinic, which I think is unique to community-based family physicians. Within the framework of the hospital-clinic collaboration (with pediatricians), we support them usually here, (or) work with schools, which I think is one of the latest topics. | 8 |
| | | <Contributing to t he growth of the team by creating a team environment> | “The lack of good performance as an entire team and as a clinic as a whole can lower the value (of the team), which makes me feel that we really need to ensure that each member will grow up.” | 13 |
| | <Experience of being able to address the medical needs of their organizations and the society in a timely manner> | <Responding to medical needs of the society> | “There is a great social demand for it (establishment of outpatient care for fever related to the COVID-19 pandemic), so I see it (establishment of providing outpatient care for fever) as very valuable.” | 2 |
| | | <Active involvement in community activities> | “Community activities are part of the responsibilities of primary care and family physicians after all. I also think they have other tasks to handle. Of course, we accepted it unconditionally (at the request of a kindergarten doctor) because it was our area. (...) We sometimes accept requests for talks at local child care salons or meetings for the elderly, saying ‘Yeah, why not?’” | 5 |
| | | <Performing situation-specific tasks at the request of the medical institution> | “Rather than choosing jobs, general practitioners work (see patients) to meet the demand of the container (institution) or the situation (of the institution or society). It’s not like, ‘Hey patients, I can do this, so come to me (if you’re having trouble with this).’ It’s more like, ‘I’ll be there to do what I’m expected to do (by the institution or society).’ That’s what a general practitioner is like, I think.” | 7 |
| | <Experience of disseminating knowledge that led to solutions to local problems> | <Involvement in research in areas closer to society> | “I think I’ll be very happy if it (my research) provides the basis for a decision to change society, even if only slightly. I think it is normal for organ-specific specialists to approach research questions arising there (their medical practice). For our (primary care physicians’) research, on the other hand, they (research questions) seem to be closer to society.” | 2 |
| | | <Studying questions arising in the workplace and communicating the results that change society> | “I collected nursing fee schedules over years from all over the country for my study on the evaluation of nursing. (...) A report on the actual practice (of nursing) in the form of formal research may lead to policy changes, which will then change the way medical practice is being delivered. Then, I analyzed and reported the results from the medical practice as larger data using numbers. This back-and-forth between medical practice and research is really interesting.” | 6 |
| | <Experience of having their studies connected to the world> | | “If I publish a paper on something, I will be asked to write an article for this or that journal, or I will receive a request for peer-review from a specialist’s point of view, like once every three days. (...) If it (the finding from my research) catches the attention of someone in the world, it is likely that that person will find an intellectual stimulus in it. That is a very rewarding part of being a researcher.” | 1 |
